# Tolerogenic β2-glycoprotein I DNA vaccine and FK506 as an adjuvant attenuates experimental obstetric antiphospholipid syndrome

**DOI:** 10.1371/journal.pone.0198821

**Published:** 2018-06-12

**Authors:** Ya-Hsuan Chao, Der-Yuan Chen, Joung-Liang Lan, Kuo-Tung Tang, Chi-Chien Lin

**Affiliations:** 1 Institute of Biomedical Science, National Chung-Hsing University, Taichung, Taiwan; 2 Division of Immunology and Rheumatology, Department of Internal Medicine, China Medical University Hospital, Taichung, Taiwan; 3 Division of Allergy, Immunology and Rheumatology, Taichung Veterans General Hospital, Taichung, Taiwan; 4 Department of Biotechnology, Asia University, Taichung, Taiwan; 5 Department of Medical Research, China Medical University Hospital, Taichung, Taiwan; Fred Hutchinson Cancer Research Center, UNITED STATES

## Abstract

DNA vaccines have recently emerged as a therapeutic agent for treating autoimmune diseases, such as multiple sclerosis. Antiphospholipid antibody syndrome (APS) is an autoimmune disease characterized by β2-glycoprotein I (β2-GPI)-targeting antiphospholipid antibodies (APAs) and vascular thrombosis or obstetrical complications. To examine the therapeutic potential of a β2-GPI DNA vaccine, we administered a vaccine mixed with FK506 as an adjuvant to a mouse model of obstetric APS. First, the pCMV3-β2-GPI DNA vaccine, which encodes the full-length human β2-GPI gene, was constructed. Then, we administered the β2-GPI DNA vaccine in 0.1 ml of saline, mixed with or without 100 μg of FK506, intramuscularly to the mice on days 28, 35 and 42. Blood titers of the anti-β2-GPI antibody, platelet counts, activated partial thromboplastin times (aPTTs), and the percentage of fetal loss were measured. We also stimulated murine splenic T cells ex vivo with β2-GPI and determined the T helper cell proportion and cytokine secretion. The administration of the β2-GPI DNA vaccine mixed with FK506 reduced the blood IgG anti-β2-GPI antibody titers and suppressed APS manifestations in mice. The combination also suppressed interferon-γ and interleukin (IL)-17A secretion but increased the Treg cell proportion and IL-10 secretion in murine splenic T cells following ex vivo stimulation with β2-GPI. Our results demonstrated the therapeutic efficacy of a β2-GPI DNA vaccine and FK506 as an adjuvant in a murine model of obstetric APS. Possible mechanisms include the inhibition of Th1 and Th17 responses and the up-regulation of Treg cells.

## Introduction

Antiphospholipid antibody syndrome (APS) is an autoimmune disease characterized by the presence of antiphospholipid antibodies (APAs, including the anti-β2-glycoprotein I (β2-GPI) antibody, anticardiolipin antibody, and lupus anticoagulant) and vascular thrombosis or obstetrical complications [1]. The main target antigen in APS is β2-GPI [2], a plasma glycoprotein that participates in a variety of physiological pathways, such as lipoprotein metabolism, coagulation and complement regulation [3,4]. It has been speculated that APAs bind to the cell membrane through β2-GPI and subsequently activate membrane receptors and downstream signal transduction [3], resulting in the activation of the complement [5–7] and the coagulation cascade. Therefore, the development of autoimmunity towards β2-GPI is critical in the pathogenesis of APS.

Investigators have demonstrated the importance of β2-GPI-specific autoreactive T cells in the pathogenesis of APS. For example, β2-GPI-specific autoreactive T cells have been found in APS patients [8]. Experiments also showed that these autoreactive T-cells stimulate B cells to produce anti-β2-GPI antibodies through interleukin (IL)-6 and CD40-CD40 ligand engagement [9]. Tomer et al. successfully suppressed experimental APS in mice using anti-CD4 monoclonal antibodies [10]. Oral low dose β2-GPI could also induce tolerance and prevent the development of APS in mice [11]. Furthermore, tolerance can be adoptively transferred to naive mice. Taken together, β2-GPI-specific autoreactive T cells appear to be a potential therapeutic target in APS.

DNA vaccines have attracted the attention of the scientific community since the early 1990s [12]. Tang and Johnston first described DNA delivery to the skin of mice using a ‘gene gun’ to deliver human growth hormone as a gene therapy [13]. Afterward, investigators considered this method useful in generating antibodies against specific transgene products. Many related studies have been conducted. However, the use of DNA vaccines in humans remains limited, despite their success in various animal models. To date, DNA vaccines are licensed for only veterinary use [14,15]. More recently, investigators have attempted to use tolerogenic DNA vaccines to treat autoimmune diseases. Fissolo et al found that a myelin oligodendrocyte glycoprotein DNA vaccine suppressed the manifestations of experimental autoimmune encephalomyelitis (EAE) in mice, both prophylactically and therapeutically [16]. Kang et al. also demonstrated that DNA vaccination, when applied with FK506 as an adjuvant, could induce antigen-specific tolerance and prevent EAE development [17]. We hypothesized that a tolerogenic DNA vaccine would be effective for treating APS. Therefore, we administered a β2-GPI DNA vaccine, with or without FK506 as an adjuvant, to a murine model of obstetric APS to examine its therapeutic potential.

## Materials and methods

### Animals and cell lines

Female BALB/c mice that were 4 weeks old were purchased from the National Laboratory Animal Center (Taipei, Taiwan). The mice were housed in cages with free access to food and water; the room was under temperature (22 ± 2°C) and humidity (45–65%) control and a 12-h light/dark cycle. This study was carried out in strict accordance with the recommendations in the Guide for the Care and Use of Laboratory Animals of the National Institutes of Health. The protocol was approved by the Committee on the Ethics of Animal Experiments of the National Chung Hsing University, Taiwan (Protocol Number: NCHU-IACUC-104-115). All surgery was performed under sodium pentobarbital anesthesia, and all efforts were made to minimize suffering. The COS-1 cell line was cultured in Dulbecco’s modified Eagle’s medium (Gibco-BRL, New York, NY) supplemented with 10% fetal bovine serum and antibiotics (100 units/ml penicillin and 100 mg/ml streptomycin, GibcoBRL).

### DNA expression vectors

**Fig 1:**
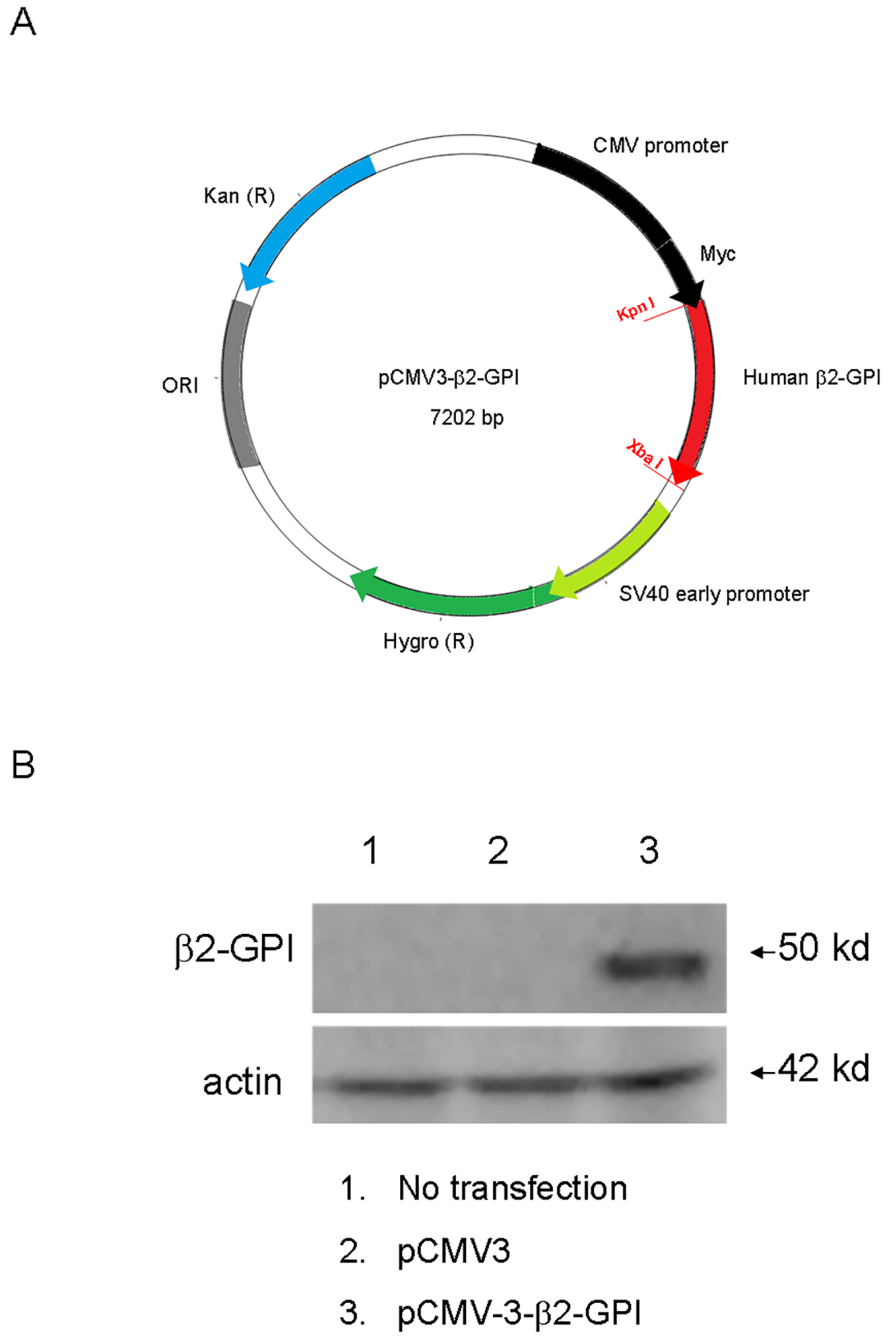
Characterization of the DNA vaccine. (A) Schematic diagram of the β2-GPI-expressing vectors. The plasmids were named “β2-GPI DNA vaccine” (B) Expression of β2-GPI in vitro. COS-1 cells were transfected with the “β2-GPI DNA vaccine” plasmids, and β2-GPI expression levels were determined by western blotting.

The pCMV3-β2-GPI plasmid, which encoded the full-length human β2-GPI gene (1038 base pairs) (Fig 1), was purchased from NovoPro (Shanghai, China). The pCMV3-β2-GPI plasmid was transfected into COS-1 cells with Lipofectamine 2000 according to the manufacturer’s instructions (Invitrogen, Carlsbad, CA). The transfected cells were harvested 24 h later, and the protein expression of β2-GPI was confirmed by Western blot using an anti-β2-GPI antibody (clone 17D11.1, Millipore, Temecula, CA). We also performed DNA sequencing and found no mutations in the β2-GPI gene.

### Establishment of a murine model of obstetric APS

Mice were immunized with β2-GPI (10 μg/mouse) (Thermo, Tewksbury, MA) in complete Freund's adjuvant (Sigma-Aldrich, St. Louis, MO). Booster immunizations with β2-GPI of 10 μg/mouse in complete Freund's adjuvant were given at 3 weeks. The mice were mated at day 42, and coital vaginal plugs, indicating successful mating, were counted in the morning during the following days. Whole blood samples were collected from the orbital sinus on day 56. Blood titers of anti-β2-GPI, platelet counts, and activated partial thromboplastin times (aPTTs) were examined. The mice were then sacrificed by asphyxiation with CO_2_; the uteri were retrieved, and the fetuses were counted [18]. The percentage of fetal loss was calculated as the number of absorbed fetuses divided by the total number of normal and absorbed fetuses.

### Determination of platelet count and aPTT

EDTA-anticoagulated blood samples were analyzed using a HEMAVET 950 hematology analyzer (Drew Scientific, Miami Lakes, FL). Blood smears were prepared and stained with the HEMAVET^®^ reagent kit. Platelet counts were then measured. Blood samples were also anti-coagulated with sodium citrate 3.2% (1: 9) and centrifuged to obtain serum samples. The aPTTs were measured by the clotting method (Sysmex CA-530, Kobe, Japan).

### Determination of blood titers of the anti-β2-GPI IgG antibody

Serum samples collected on day 56 were diluted in Tris-buffered saline (1: 100, pH 8.0) that contained 1% BSA and 0.5% Tween-20. Anti-β2-GPI IgG concentrations were measured with an ELISA kit (MyBioSource, San Diego, CA) according to the manufacturer's protocol. Although the anti-domain I anti-β2-GPI antibody is now recognized as the main pathogenic subset of antiphospholipid antibodies, a proportion of APS patients positive for the anti-β2-GPI antibody are negative for anti-domain I IgG [19,20]. Therefore, we did not measure the anti-domain I anti-β2 glycoprotein I antibody in our experiments.

### DNA vaccination

The pCMV-3 mock plasmid and pCMV3-β2-GPI plasmid were prepared as described previously [21]. The mice were injected in their hind leg muscles three times (on days 28, 35, and 42) with the DNA plasmid (100 μg), FK506 (10 μg) or a mixture of both the DNA plasmid and FK506 in 100 μl of saline.

### Cell proliferation assay

Splenocytes were extracted from the mice on day 56 and cultured for 96 h at 2× 10^5^ cells/well into 96-well plates with 10 μg/ml β2-GPI. Cells were pulsed with 1 μCi/well of [^3^H]-thymidine (MP Biomedicals, Solon, OH) followed incubation for an additional 4h. Splenocytes were then harvested and the incorporation of radioactivity measured.

### Cytokine production analysis

Splenocytes were extracted from the mice on day 56 and cultured at 2× 10^6^ cells/well into 24-well plates. Supernatants were collected after 96 h culture with 10 μg/ml β2-GPI. Cytokine concentrations, including mouse interferon (IFN)- γ (cat. 88–8314), IL-17A (cat. 88-7371-88), IL-4, (cat. 88-7044-88) IL-10 (cat. 88-7105-88) and transforming growth factor (TGF)- β (cat. 88-8350-88) (eBioscience, San Diego, CA), were measured by standard sandwich ELISA according to the manufacturer's protocol.

### Intracellular staining

To analyze T cell subtypes, splenocytes were extracted from the mice on day 56 and stimulated with 10 μg/ml β2-GPI for 96 h. Golgistop solution (BD Biosciences, San Diego, CA) was added to the culture 6 h prior to cell harvesting. The cells were washed twice with the FACScan buffer and stained with a phycoerythrin (PE)-conjugated anti-mouse CD4 antibody (clone RM4-5, Biolegend San Diego, CA). The cells were then fixed and intracellularly stained with the Cytofix/Cytoperm Plus Kit (BD Biosciences, San Diego, CA) according to the manufacturer's instructions. FITC-conjugated mAbs specific to murine IFN-γ (clone XMG1.2), IL-4 (clone 11B11), IL-17A (clone TC11-18H10.1) and Foxp3 (clone 150D) were purchased from BioLegend. All samples were analyzed with an Accuri C5 cytometer using the C6 Accuri system software (BD Biosciences, San Jose, CA).

### Statistical analysis

Statistical analyses were performed using GraphPad Prism (version 5 for Windows; GraphPad Software). All quantitative data were presented in the form of mean and the standard deviation unless specified otherwise. Mann-Whitney U test was used to compare data between two groups. A two-tailed p value of < 0.05 was considered statistically significant.

## Results

### The impact of the β2-GPI DNA vaccine on APS manifestations

In our first experiment, we tested the effects of the β2-GPI DNA vaccine on obstetric APS. As shown in Table 1, the immunization of saline-treated mice with β2-GPI/complete Freund's adjuvant (CFA) (control APS group) led to a reduced platelet count, prolonged aPTT, and increased fetal loss when compared with the non-β2-GPI immunized mice (normal group). In contrast, no significant differences in the manifestations of APS were found in APS mice treated with saline, mock DNA vector, or β2-GPI DNA vaccine. Our findings suggested that the β2-GPI DNA vaccination alone had no effects on APS.

**Table 1:** Clinical manifestations in mice.

Mouse groups^a^				
	Normal group	Control APS group	Mock DNA vector-treated APS group	β2-GPI DNA vaccine-treated APS group
aPTT (seconds)	29.4 ± 2.7**	83.7 ± 14.8	77.4 ± 24.3 ^ns^	67.4 ± 23.6 ^ns^
Platelet count (×10^3^ cells/mm^3^)	684 ± 219*	264 ± 144	244 ± 147^ns^	338 ± 176^ns^
% Fetal loss	10 ± 9***	41 ± 19	45 ± 16 ^ns^	36 ± 18^ns^

The data are presented as the means ± standard deviation of triplicate assays from 6–8 mice/group.

^ns^p>0.05,

*p<0.05,

**p< 0.01,

***p< 0.001 versus the control APS group.

^a^normal group, β2-GPI non-immunized mice; control APS group, saline-treated APS mice; mock DNA vector-treated APS group, mock plasmid-treated APS mice; β2-GPI DNA vaccine-treated APS group, β2-GPI DNA vaccine-treated APS mice.

APS, antiphospholipid syndrome; aPTT, activated partial thromboplastin time.

### The impact of the mixture of the β2-GPI DNA vaccine and FK506 on APS manifestations

To test the effects of immunomodulator-augmented DNA tolerogenic vaccination, we treated mice with a mixture of the β2-GPI DNA vaccine and FK506. As shown in Table 2, the administration of either the β2-GPI DNA vaccine or FK506 alone did not ameliorate APS manifestations. However, the β2-GPI DNA vaccine administered with FK506 (β2-GPI DNA vaccine/FK506 group) did significantly suppress APS manifestations, such as a prolonged aPTT, decreased platelet count and increased percentage of fetal loss.

**Table 2:** Clinical manifestations in mice.

Mouse groups^a^					
	Normal group	Control APS group	FK506-treated APS group	β2-GPI DNA vaccine-treated APS group	β2-GPI DNA vaccine/FK506-treated APS group
aPTT (seconds)	24.7 ± 7.9**	88.8 ± 12.1	81.0 ± 19.3^ns^	65.1 ± 24.4^ns^	51.6 ± 25.5*
Platelet count (×10^3^ cells/mm^3^)	664 ± 288**	230 ± 123	250 ± 147^ns^	273 ± 96^ns^	491 ± 208*
% Fetal loss	8±8***	40 ± 12	41 ± 16^ns^	38 ± 14^ns^	25 ± 18*

The data are presented as the means ± standard deviation of triplicate assays from 8–12 mice/group.

^ns^p>0.05,

*<0<0.05,

**p< 0.01,

***p< 0.001 versus the control APS group.

^a^normal group, β2-GPI non-immunized mice; control APS group, saline-treated APS mice; FK506-treated APS group, FK506-treated APS mice; β2-GPI DNA vaccine-treated APS group, β2-GPI DNA vaccine-treated APS mice; β2-GPI DNA vaccine/FK506-treated APS group, APS mice treated with the β2-GPI DNA vaccine mixed with FK506.

APS, antiphospholipid syndrome; aPTT, activated partial thromboplastin time.

### The impact of the mixture of the β2-GPI DNA vaccine and FK506 on murine splenic T cell proliferation and cytokine secretion after β2-GPI stimulation ex vivo

**Fig 2:**
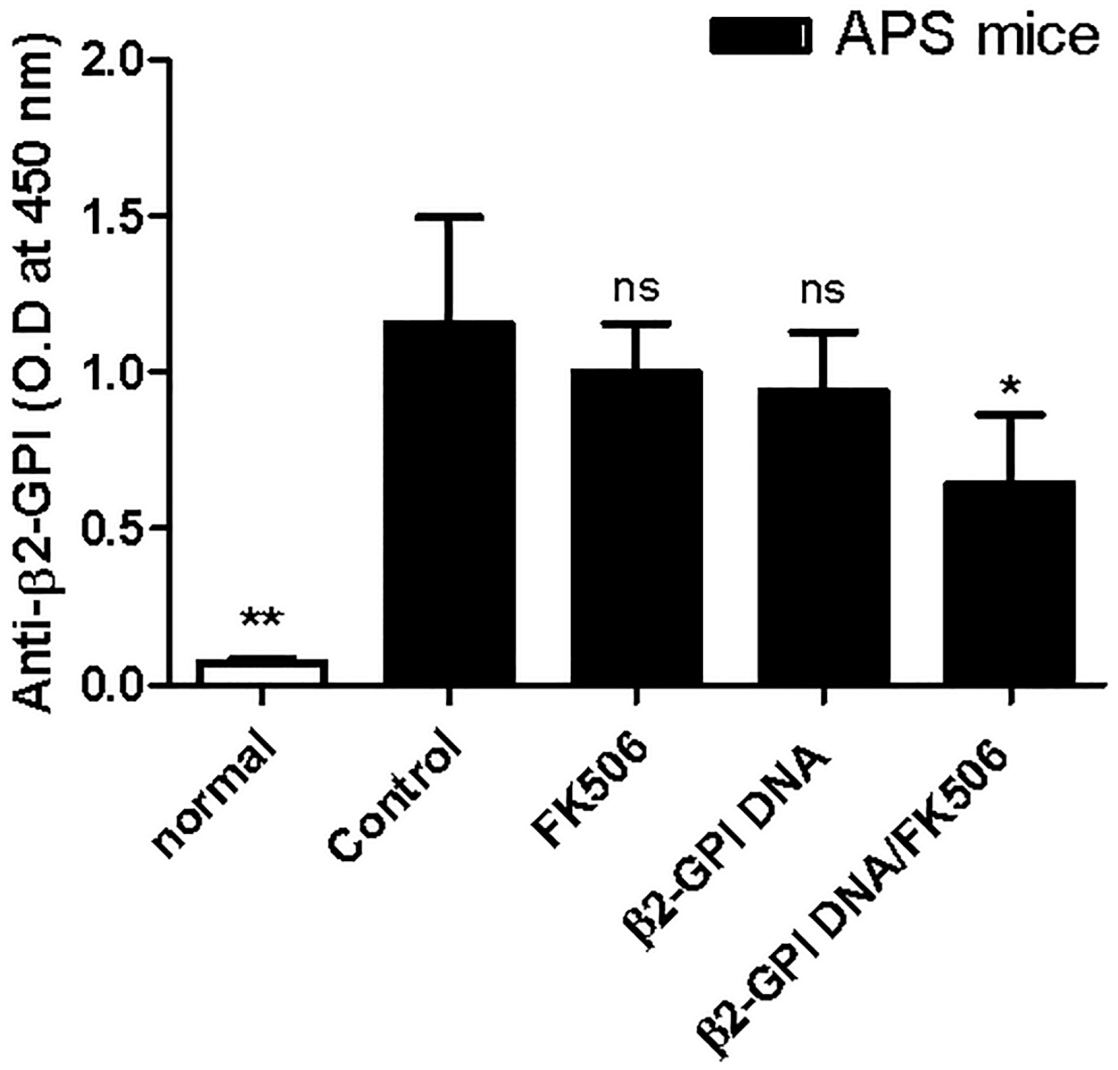
Effects of the β2-GPI DNA vaccine and FK506 treatment on IgG anti-β2-GPI antibody levels. Serum samples were obtained from each mouse group on day 56, and IgG anti-β2-GPI antibody levels were analyzed by ELISA. The data are presented as the means ± standard deviation of triplicate assays from six mice/group. ^ns^p>0.05, *p < 0.05, ** p < 0.01 versus the control APS group.

**Fig 3:**
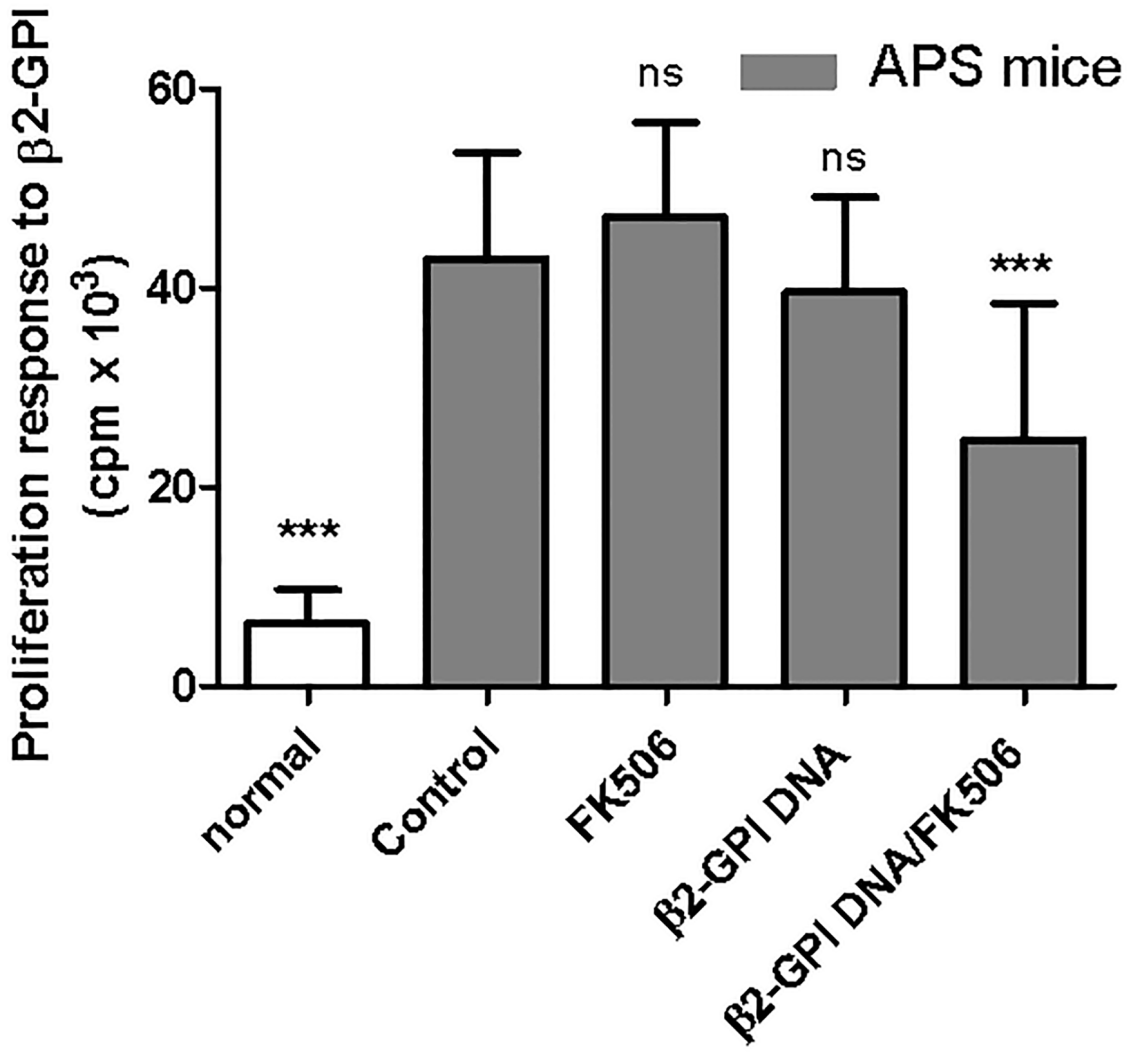
Effects of the β2-GPI DNA vaccine and FK506 treatment on β2-GPI-specific spleen cell proliferation. Spleen cells were purified from the different mouse groups on day 56 and stimulated with recombinant β2-GPI protein (10 μg/mL). After 96 h, cell proliferation was assessed by [^3^H]-TdR incorporation. The results are presented as the means ± standard deviation of triplicate assays from six mice/group. ^ns^p>0.05, *** p < 0.001 versus the control APS group.

Both autoantibody and T cell responses are important in APS pathogenesis [3,22,23]. To further test the tolerogenic effects of the mixture of the β2-GPI DNA vaccine and FK506, we determined the serum titers of APA and the proliferation of splenic β2-GPI-specific T cells. Consistent with previous studies, high serum anti-β2-GPI IgG titers were detected in all APS mice; however, the IgG titers were significantly lower in the β2-GPI DNA vaccine/FK506-treated APS group (Fig 2). In addition, β2-GPI immunization increased the proliferation of splenic T cells in response to β2-GPI stimulation, whereas treatment with the β2-GPI DNA vaccine/FK506 significantly suppressed this proliferation response (Fig 3).

**Fig 4:**
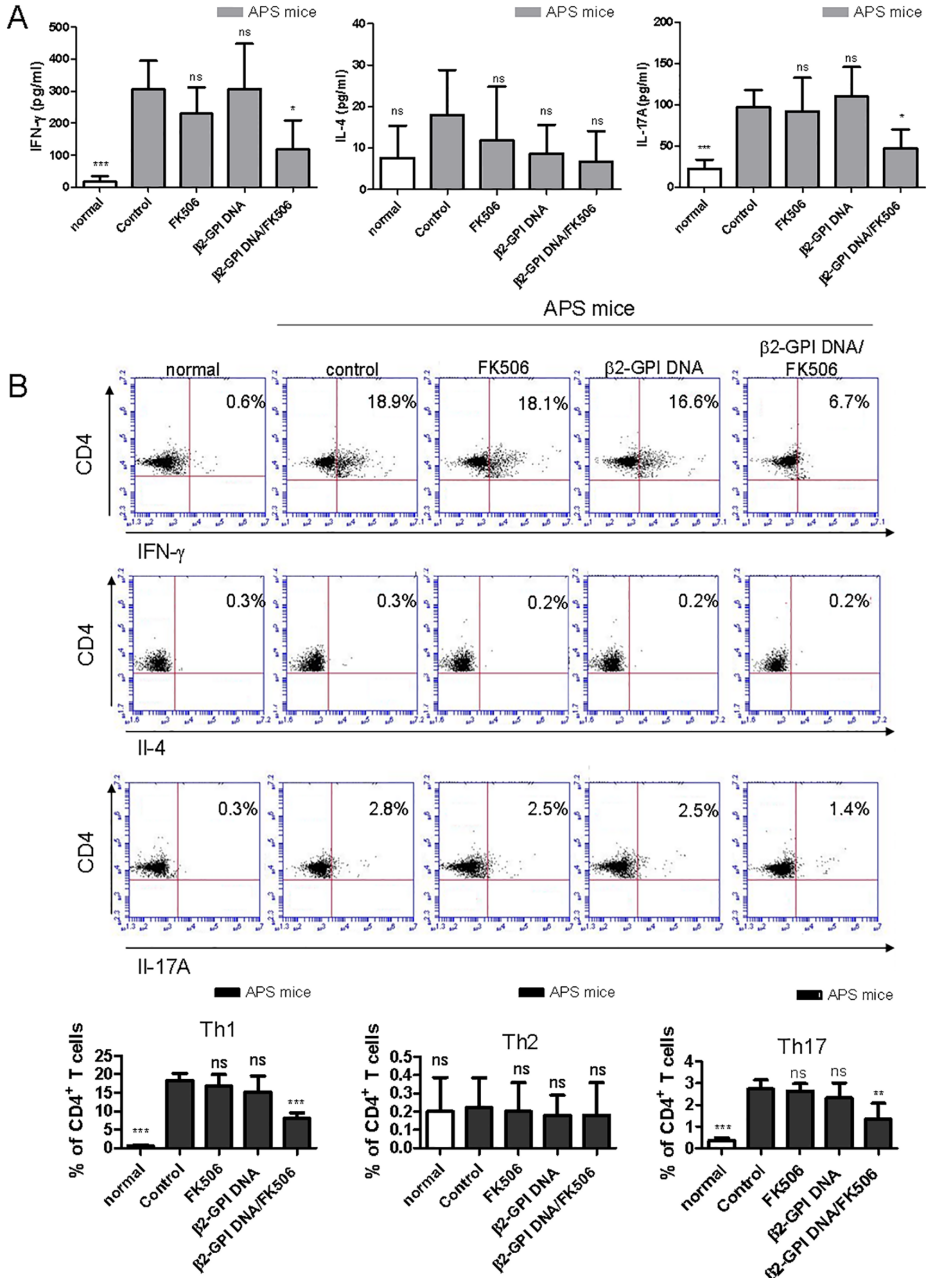
Effects of the β2-GPI DNA vaccine and FK506 treatment on cytokine production. Spleen cells were purified from the different mouse groups on day 56 and stimulated with recombinant β2-GPI protein (10 μg/mL). After 96 h, (A) the culture supernatants were collected, and interferon (IFN)- γ, interleukin (IL)-4 and IL-17A production was analyzed in triplicate by ELISA. The data are presented as the means ± standard deviation (SD) of triplicate assays from six mice/group. (B) The percentages of IFN-γ-expressing CD4+ T cells, IL-4-expressing CD4+ T cells and IL-17A-expressing CD4+ T cells were determined by flow cytometry. The dot plot shows data from one representative mouse from each group. The bar graph represents the mean ± SD of six mice from three independent experiments. ^ns^p>0.05, *p < 0.05, **p < 0.01 versus the control APS group.

Cytokines play critical roles in immune polarization. To study the cytokine profiles in the spleen after the induction of APS, the secretion and expression of IFN-γ, IL-4 and IL-17A after β2-GPI stimulation were determined for splenic T cells using the ELISA technique and intracellular staining analyzed by flow cytometry. As shown in Fig 4A, only the levels of IFN-γ and IL-17A, but not IL-4, in the culture medium were significantly lower in the β2-GPI DNA vaccine/FK506 treatment group than in the control APS group. In addition, the intracellular cytokine staining results showed that the β2-GPI DNA vaccine/FK506-treated mice had fewer IFN-γ+CD4+ (Th1) and IL-17A+CD4+ T cells (Th17) than the other groups of mice (Fig 4B). Regarding IL-4+CD4+ (Th2) cells, we found no significant differences among all groups of mice (Fig 4B). Taken together, our results suggested that β2-GPI DNA vaccine/FK506 treatment could suppress β2-GPI-specific humoral (IgG anti-β2-GPI antibody) and T cell immune responses (Th1 and Th17) in APS mice.

### The impact of the mixture of the DNA vaccine and FK506 on the regulatory T cell proportion and anti-inflammatory cytokine secretion in murine splenic T cells after β2-GPI stimulation ex vivo

**Fig 5:**
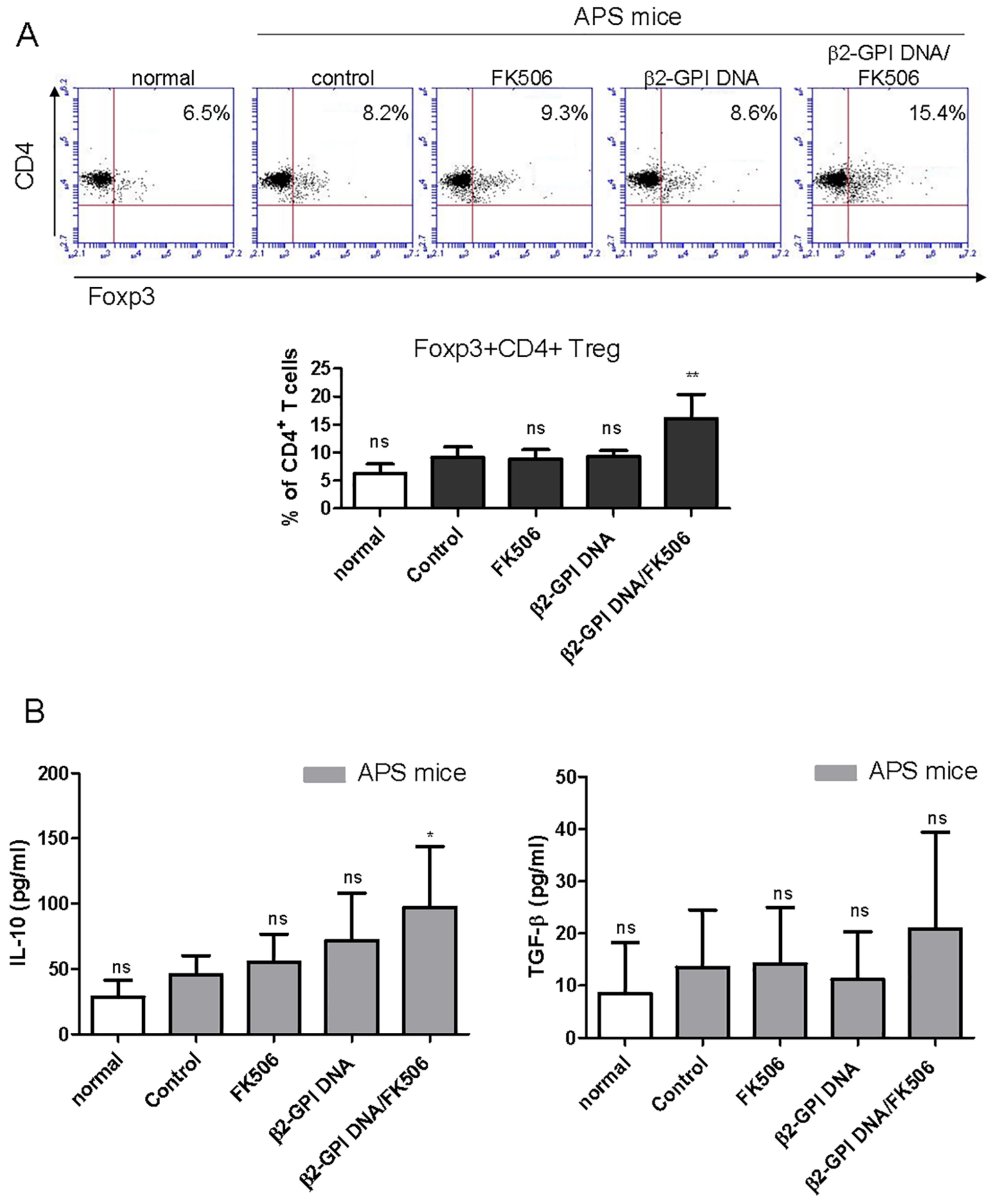
Effects of the β2-GPI DNA vaccine and FK506 treatment on β2-GPI-specific Treg cell response. Spleen cells were purified from the different mouse groups on day 56 and stimulated with recombinant β2-GPI protein (10 μg/mL). After 96 h, (A) the percentage of Foxp3-expressing CD4+ T cells was determined by flow cytometry. The dot plot shows data from one representative mouse from each group. The bar graph represents the mean ± standard deviation (SD) of six mice from three independent experiments. (B) The culture supernatants were collected, and IL-10 and TGF-β production was analyzed in triplicate by ELISA. The data are presented as the means ± SD of triplicate assays from six mice/group. ^ns^p>0.05, **p < 0.01 versus the control APS group.

Since the β2-GPI DNA vaccine/FK506 significantly inhibited the response of β2-GPI-specific T cells, we further determined whether the tolerance induction was associated with Treg cell expansion. As shown in Fig 5A, treatment with the β2-GPI DNA vaccine or FK506 alone had no effects on the proportion of Foxp3+CD4+ Treg cells in the splenic T cells after β2-GPI stimulation. In contrast, the β2-GPI DNA vaccine mixed with FK506 significantly increased the proportion of Foxp3+CD4+ Treg cells. The secretion of the anti-inflammatory cytokines IL-10 and TGF-β by splenic T cells after β2-GPI stimulation was also enhanced by β2-GPI DNA vaccine/FK506 treatment (Fig 5B), although this change was statistically insignificant for TGF-β (Fig 5B). Together, these data suggested that the reduction in the β2-GPI-specific T helper response after β2-GPI/FK506 treatment was, at least in part, caused by an increase in Treg cells.

## Discussion

This is the first study on the therapeutic potential of a tolerogenic DNA vaccine in a murine model of obstetric APS. Our results demonstrated that a DNA vaccine mixed with FK506 as an adjuvant could suppress the manifestations of APS in a murine model. This effect is likely mediated through the suppressed response of the β2-GPI-specific humoral and cellular T helper cells and the increased number of Treg cells.

DNA vaccines are beneficial because of their flexibility, stability, easy storage and low cost [12]. The therapeutic failure of DNA vaccines in large animals and humans is partly related to their low immunogenicity. After intramuscular injection, plasmid uptake by myocytes is often unpredictable. Furthermore, the cross-presentation of gene products of the DNA vaccine by antigen-presenting cells may be insufficient to elicit the immune response. On the other hand, an insufficient pro-inflammatory cytokine milieu after DNA vaccination may lead to the development of immune tolerance [24]. Previous studies have reported the skewing of T helper responses [25] or induction of IL-10-producing type 1 Treg cells [26] after DNA vaccination; these effects inhibit the development of autoimmunity. In fact, the therapeutic efficacy of DNA vaccines has been demonstrated in animal models for a number of autoimmune diseases, such as rheumatoid arthritis [27], insulin-dependent diabetes mellitus [28] and multiple sclerosis (MS) [16]. Moreover, clinical trials of DNA vaccines in diabetes mellitus [29] and MS patients have shown some preliminary efficacy [30, 31]. Roep et al. enrolled 80 type 1 diabetes patients and found that a DNA plasmid encoding proinsulin (BHT-3021) decreased the number of proinsulin-reactive CD8+ T cells in the peripheral blood and preserved pancreatic β cell function [29]. Garren et al. conducted a phase II trial of 267 relapsing-remitting MS patients who received BHT-3009, a DNA vaccine encoding the human myelin basic protein [31]. The authors observed a trend towards a reduction in new enhanced brain magnetic resonance imaging lesions when compared with the placebo group. In line with these findings, it is not surprising that a DNA vaccine and FK506 as an adjuvant could be therapeutically effective against APS, an autoimmune disease with a distinct autoantigen, “β2-GPI” [3]. Further trials are needed to demonstrate the efficacy in humans.

FK506 is an immunosuppressant widely used for treating autoimmune disease [32,33] and minimizing transplant rejection [34]. FK506 suppresses T cell activation via inhibiting the calcineurin-nuclear factor of activated T cells (NFAT) pathway, as well as the c-Jun N-terminal kinase (JNK)-p38 pathway [35]. Its pharmacological actions are similar to those of cyclosporine, but FK506 has a better cardiovascular risk profile and lower nephrotoxicity [36]. Although FK506 fails to induce tolerogenic dendritic cells (DCs) according to some investigators [37,38], it has been demonstrated by others to affect the growth and antigen presentation of DCs [39, 40], as well as DC-T cell interactions [41]. In addition, FK506 has been shown to inhibit conventional T cell proliferation and promote Treg proliferation [42]; thus, it could create a local tolerogenic environment when used together with the DNA vaccine. In the EAE model, FK506 successfully induced antigen-specific tolerance and prevented the autoimmune disease when used as an adjuvant for the DNA vaccine [17]. In line with this, our results also note the utility of FK506 as an adjuvant for DNA vaccines in treating autoimmune diseases.

DNA vaccines exert their therapeutic effects on autoimmune diseases presumably through various mechanisms, such as down-regulating Th1 immune responses [43], increasing Th2 responses [25], inducing Treg cells [16,44], and up-regulating IFN-β [45]. In our study, treating APS mice with a mixture of the DNA vaccine and FK506 decreased Th1 and Th17 responses and increased the proportion of Foxp3+CD4+ Treg cells in splenic T cells after β2-GPI stimulation. At the same time, the secretion of the anti-inflammatory cytokine IL-10 by splenic T cells after β2-GPI stimulation was also increased. Interestingly, increased Th1 and Th17 responses [22,46] and decreased Treg expression [47] have been speculated to be involved in the pathogenesis of APS. The therapeutic efficacy of DNA vaccine/FK506 treatment is probably the result of multiple mechanisms.

In conclusion, we have demonstrated the therapeutic efficacy of a β2-GPI DNA vaccine mixed with FK506 as an adjuvant in a murine model of obstetric APS. The possible mechanisms include inhibited anti-β2-GPI antibody production, reduced β2-GPI-specific Th1/Th17 responses, and up-regulated Treg responses. However, our results cannot be extrapolated to vascular APS due to potentially different pathogenic mechanisms.

## Supporting information

S1 Fig Raw data for Fig 2. (PDF)

S2 Fig Raw data for Fig 3. (PDF)

S3 Fig Raw data for Fig 4A. (PDF)

S4 Fig Raw data for Fig 4B. (PDF)

S5 Fig Raw data for Fig 5A. (PDF)

S6 Fig Raw data for Fig 5B. (PDF)

S1 Table Raw data for Table 1. (PDF)

S2 Table Raw data for Table 2. (PDF)
