# Supplementary material for: Tolerogenic β2-glycoprotein I DNA vaccine and FK506 as an adjuvant attenuates experimental obstetric antiphospholipid syndrome
Source: PLoS One. 2018 Jun 12;13(6):e0198821. doi: 10.1371/journal.pone.0198821 (PMC5997307; doi:10.1371/journal.pone.0198821)
Supplement: S2 Fig — (PDF) [file pone.0198821.s002.pdf]

tion response t

Normal	Control APS	FK506/APS	B2-GPI DNA/APS	B2-GPI DNA+FK506/APS
5.5	43.5	45.6	44.2	22.6
4.6	41.4	66.2	51.2	40
1.3	25.6	43.5	39.2	12.4
6.7	51.5	41	44.2	31.4
8.5	39.5	44.6	35	29.6
11.2	56.2	41.5	23.6	12
