# Supplementary material for: Tolerogenic β2-glycoprotein I DNA vaccine and FK506 as an adjuvant attenuates experimental obstetric antiphospholipid syndrome
Source: PLoS One. 2018 Jun 12;13(6):e0198821. doi: 10.1371/journal.pone.0198821 (PMC5997307; doi:10.1371/journal.pone.0198821)
Supplement: S3 Fig — (PDF) [file pone.0198821.s003.pdf]

Th1

Normal	Control APS	FK506/APS	B2-GPI DNA/APS	B2-GPI DNA+FK506/APS
0.6	18.9	18.1	16.6	6.8
0.3	20.3	16.2	16.1	6.6
1	17.3	19.3	10.76	7.3
0.4	15.6	11.6	21.2	10.4
0.3	19.6	18.6	10.8	8.7

Th2

Normal	Control APS	FK506/APS	B2-GPI DNA/APS	B2-GPI DNA+FK506/APS
0.3	0	0.4	0.2	0
0	0.4	0.1	0.3	0
0	0.3	0	0.2	0.3
0.4	0.3	0.3	0.2	0.4
0.3	0.1	0.2	0	0.2

IL-17A

Normal	Control APS	FK506/APS	B2-GPI DNA/APS	B2-GPI DNA+FK506/APS
11	109	109	124	73
21	81	87	109	12
11	109	67	166	51
28	122	109	120	44
23	98	33	78	71
41	67	151	66	31
