# Supplementary material for: Tolerogenic β2-glycoprotein I DNA vaccine and FK506 as an adjuvant attenuates experimental obstetric antiphospholipid syndrome
Source: PLoS One. 2018 Jun 12;13(6):e0198821. doi: 10.1371/journal.pone.0198821 (PMC5997307; doi:10.1371/journal.pone.0198821)
Supplement: S4 Fig — (PDF) [file pone.0198821.s004.pdf]

Th1

Normal	Control APS	FK506/APS	B2-GPI DNA/APS	B2-GPI DNA+FK506/APS
0.6	18.9	18.1	16.6	6.8
0.3	20.3	16.2	16.1	6.6
1	17.3	19.3	10.76	7.3
0.4	15.6	11.6	21.2	10.4
0.3	19.6	18.6	10.8	8.7

Th2

Normal	Control APS	FK506/APS	B2-GPI DNA/APS	B2-GPI DNA+FK506/APS
0.3	0	0.4	0.2	0
0	0.4	0.1	0.3	0
0	0.3	0	0.2	0.3
0.4	0.3	0.3	0.2	0.4
0.3	0.1	0.2	0	0.2

Th17

Normal	Control APS	FK506/APS	B2-GPI DNA/APS	B2-GPI DNA+FK506/APS
0.2	2.8	2.5	2.5	1.4
0.5	3.4	3	3.3	0.6
0.4	2.3	3	2.2	0.6
0.4	2.6	2.3	2.2	2
0.3	2.5	2.4	1.4	2.1
