# Supplementary material for: Tolerogenic β2-glycoprotein I DNA vaccine and FK506 as an adjuvant attenuates experimental obstetric antiphospholipid syndrome
Source: PLoS One. 2018 Jun 12;13(6):e0198821. doi: 10.1371/journal.pone.0198821 (PMC5997307; doi:10.1371/journal.pone.0198821)
Supplement: S5 Fig — (PDF) [file pone.0198821.s005.pdf]

Foxp3+cd4+	Normal	Control APS	FK506/APS	B2-GPI DNA/APS	B2-GPI DNA+FK506/APS
	10.9	12	11.3	10.8	23.8
	9.8	11.9	11.2	12.5	22.1
	9.1	8.9	8.76	8.8	12.7
	8.9	9.7	9.3	10.2	23.9
	6.8	10.9	9.9	8.8	14.9
