# Supplementary material for: Tolerogenic β2-glycoprotein I DNA vaccine and FK506 as an adjuvant attenuates experimental obstetric antiphospholipid syndrome
Source: PLoS One. 2018 Jun 12;13(6):e0198821. doi: 10.1371/journal.pone.0198821 (PMC5997307; doi:10.1371/journal.pone.0198821)
Supplement: S6 Fig — (PDF) [file pone.0198821.s006.pdf]

IL-10

Normal	Control APS	FK506/APS	B2-GPI DNA/APS	B2-GPI DNA+FK506/APS
12	65	76	41	183
33	55	42	112	109
39	24	51	41	98
41	41	33	51	54
33	51	87	61	73
12	39	41	123	66

TGF-beta

Normal	Control APS	FK506/APS	B2-GPI DNA/APS	B2-GPI DNA+FK506/APS
12	16	12	12	14
0	0	33	0	0
0	24	11	0	0
18	0	0	16	36
21	17	12	21	41
0	24	17	18	34
