# Supplementary material for: Tolerogenic β2-glycoprotein I DNA vaccine and FK506 as an adjuvant attenuates experimental obstetric antiphospholipid syndrome
Source: PLoS One. 2018 Jun 12;13(6):e0198821. doi: 10.1371/journal.pone.0198821 (PMC5997307; doi:10.1371/journal.pone.0198821)
Supplement: S1 Table — (PDF) [file pone.0198821.s007.pdf]

aPTT (seconds)

Normal	Control APS	MOCK DNA/APS	B2-GPI DNA/APS
28.4	80	70.4	32.6
29.5	89.2	36.3	76.3
27.3	67.4	104.2	81.3
26.2	69.8	99.1	99.4
33.5	107.6	81.2	36.6
31.6	88.2	73.3	54.1

late count (x10^3 cell/mm

Normal	Control APS	MOCK DNA/APS	B2-GPI DNA/APS
612	323	526	617
983	252	201	130
905	122	278	412
612	512	133	300
397	132	165	176
596	245	163	366

Fetal loss

Normal	Control APS	MOCK DNA/APS	B2-GPI DNA/APS
0.00%	40.00%	71.43%	60.00%
16.67%	33.33%	33.33%	33.33%
16.67%	20.00%	40.00%	20.00%
16.67%	66.67%	40.00%	66.67%
16.67%	50.00%	42.86%	25.00%
0.00%	20.00%	33.33%	33.33%
0.00%	66.67%	71.43%	33.33%
16.67%	33.33%	33.33%	20.00%
