# Supplementary material for: Tolerogenic β2-glycoprotein I DNA vaccine and FK506 as an adjuvant attenuates experimental obstetric antiphospholipid syndrome
Source: PLoS One. 2018 Jun 12;13(6):e0198821. doi: 10.1371/journal.pone.0198821 (PMC5997307; doi:10.1371/journal.pone.0198821)
Supplement: S2 Table — (PDF) [file pone.0198821.s008.pdf]

aPTT (seconds)	Normal	Control APS	FK506/APS	B2-GPI DNA/APS	B2-GPI DNA+FK506/APS
	34	102.4	67.9	54.1	38.4
	25.6	89.2	88.9	55.6	51.2
	21.5	80.6	79.4	96.3	22.5
	22.3	70.4	108.5	95.2	76.3
	12.6	89.4	52.5	38.9	33.6
	32.4	100.6	88.6	51	87.6

plate count (x10^3 cell/mm^3)	Normal	Control APS	FK506/APS	B2-GPI DNA/APS	B2-GPI DNA+FK506/APS
	1094	437	465	311	509
	434	181	39	261	765
	634	130	294	221	166
	714	171	144	361	487
	829	323	245	114	631
	281	140	310	368	387

Fetal loss	Normal	Control APS	FK506/APS	B2-GPI DNA/APS	B2-GPI DNA+FK506/APS
	0.00%	50.00%	33.33%	42.86%	11.11%
	16.67%	28.57%	33.33%	28.57%	57.14%
	16.67%	25.00%	16.67%	14.29%	27.27%
	16.67%	50.00%	42.86%	33.33%	54.55%
	14.29%	57.14%	71.43%	44.44%	12.50%
	0.00%	33.33%	44.44%	55.56%	20.00%
	0.00%	33.33%	42.86%	60.00%	14.29%
	0.00%	33.33%	33.33%	42.86%	9.09%
	11.11%	50.00%	57.14%	28.57%	22.22%
